# Letter from New York

**Published:** 1881-08-20

**Authors:** Jas. H. Low

**Affiliations:** 101 East 65th St., New York City


### *LETTER FROM NEW YORK.*

The following is an extract from a letter addressed to the managing editor of this Journal:

"In visiting this great metropolis, and while traversing the portions of the city where there are so many tenement houses located, and each apartment densely inhabited by so many illy fed and poorly clad women and children, bad sewerage and poor ventilation, you are constrained to ask yourself what becomes of these people when they are taken sick? Well, they are cared for by the many self-sacrificing people who, with tender feelings accompanied by rare sympathies, administer to their wants—which is shown by the many dispensaries and hospitals with which New York city abounds, where these unfortunates can procure medicine and medical aid gratuitously.

I have visited many of these institutions, and the women's hospital is the finest of the kind in America. In charge of this hospital are four or five physicians of celebrity and distinction. I have seen several of them operate, but I can say of a truth that Dr. Emmet, as an operator, cannot be excelled in this city, nor do I believe in the world. The Doctor is polite, and is very regardful of physicians visiting the city. His corps of assistants are very gentlemanly. I believe the Doctor is an exclusive gynecologist, and may be termed a specialist. I have read with great satisfaction his late work, and his treatment in inflammation with hot water, and his insisting so much upon its usefulness in practice is novel, but nevertheless true.

The July number of your journal is received, and under the head of "Original and Selected Articles" from your numerous writers who so ably contribute to that department, many of whom evince a high mental culture and medical attainment, I cull, not unfrequently, practical and instructive ideas. And also the Abstracts, Gleanings and Formulæ specially commend themselves to the medical profession, furnishing some of the best remedies in the treatment of diseases. I have used with marked success the tartaric acid in some aggravated cases of diphtheria, together with other remedies recommended under this heading. Yours truly,

JAS. H. LOW, M. D.

101 East 65th St., New York City.

